# Supplementary material for: Use of Complementary and Alternative Therapies in People with Inflammatory Bowel Disease
Source: Int J Environ Res Public Health. 2024 Aug 28;21(9):1140. doi: 10.3390/ijerph21091140 (PMC11431664; doi:10.3390/ijerph21091140)
Supplement: Supplementary file 1 [file ijerph-21-01140-s001.zip › ijerph-3145588-supplementary.pdf]

Supplementary Table S1. Survey questions

Please select the following criteria if they apply:

- I have been diagnosed with Inflammatory Bowel Disease (either ulcerative colitis or Crohn's Disease) and am at least 18 years of age
- Alternatively, you may exit the survey now if you are not eligible OR you do not wish to participate

---

Q2 This section of the survey consists of questions about you.

Q3 What is your age now? (Select one)

- 18 - 24 years
- 25 - 34 years
- 35 - 44 years
- 45 - 54 years
- 55 years and over
- I prefer not to answer

Q4 What is your gender ? (Select one)

- male
- female
- non binary
- other __________________
- I prefer not to answer

Q5 What age were you when diagnosed with Inflammatory Bowel Disease (IBD) ?

- o (Please state) ____

Q6 What type of IBD do you have ? (Select one)

- Ulcerative colitis
- Crohn's Disease
- Other (please specify) ____

Q7 What is the highest level of education that you have completed? (Select one)

- High school
- Bachelor Degree
- Trade qualification
- Masters Degree
- PhD
- Other (please specify)

Q8 Which country do you live in ? (select one)

- Australia
- Outside of Australia (Please specify which country __________________________________

Q9 How confident are you filling out forms by yourself ?

- Extremely
- Quite a bit
- Somewhat
- A little bit
- Not at all

Q10. How often do you have someone help you read hospital materials ?

- Never
- Occasionally
- Sometimes
- Often
- Always

Q11. How often do you have problems learning about your medical condition because of difficulty reading hospital materials ?

- Never
- Occasionally
- Sometimes
- Often
- Always

Q12.Have you had surgery for your IBD ? (select one)

- Yes
- No
- Unsure

Q13 What medicine do you currently take for your IBD ? select as many as required

- Antibiotics eg ciprofloxacin, metronidazole
- Steroids eg predinsone
- 5 ASA eg sulfasalazine, mesalamine
- Immunosuppressants eg azathioprine, methotrexate
- Biological therapy eg infliximab
- None
- Other (please specify)

Q14 This question relates to the past 12 months. Have you ever seen any of the following health care providers in the last 12 months (Select as many as required)

- General Practioner
- Specialist Doctor eg gastroenterologist (please specify) ________________________________
- Chiropractor
- Homeopath
- Acupuncturist
- Herbalist
- Spiritual healer
- Nutritionist
- Dietitian
- Manipulation
- Homeopathy
- Acupuncture
- Other (please specify) ____
- Other (please specify) ____

Display This Question:
If yes to any practioner in Q14

Q15 How many times have you seen this health care provider in the past 3 months ?

- Weekly
- Fortnightly
- Monthly
- Other (please specify) ____

Display This Question:
If yes to any practioner in Q14

Q15 Please indicate the main reason you last saw the health care provider? (Select only one)

- For an acute illness/ condition that lasted less than a month
- To treat a long term health condition (one that lasted more than one month) or the symptoms from this long term condition
- To improve wellbeing
- Other (please specify) ____

Display This Question:
If yes to any Practioner in Q14

Q15 How helpful was it for you to see this health care provider ?

- Very helpful
- Somewhat helpful
- Not at all helpful
- Don't know
- Unsure
- Other comment ____-

Q18 This question relates to the past 12 months. Have you ever used any of the following complimentary or alterative medicines? (Select as many as required)

- Herbs / herbal medicine – please specify ______________________________
- Vitamins - please specify ______________________________
- Minerals- please specify ______________________________
- Other nutritional supplements- please specify ______________________________
- Homeopathic remedies please specify ______________________________
- Other supplements- please specify ______________________________

Display This Question:
If yes to any product in Q18

Q19 Do you currently use this product ?

- Yes
- No
- Other (please specify) ____

Display This Question:
If yes to any product in Q18

Q20 Please indicate the main reason you used this product ? (Select only one)

- For an acute illness/ condition that lasted less than a month
- To treat a long term health condition (one that lasted more than one month) or the symptoms from this long term condition
- To improve wellbeing
- Other (please specify) ____

Display This Question:
If yes to any product in Q18

Q21 How helpful did you find this product ?

- Very helpful
- Somewhat helpful
- Not at all helpful
- Don't know
- Unsure
- Other comment ____-

Q22 Do you have any other comments you wish to provide us about complimentary or alternative medicine use for people with IBD ? This may include any comments you may have on your experiences discussing these with health professionals

____

Q23 What is your main source of information about treatment and management of IBD ?

- My Doctor or medical specialists
- News articles
- Brochures
- Websites (please specify)
- Social media (please specify)
- Other people with IBD
- Support groups for people with IBD eg Crohns and Colitis Australia
- Journal articles
- Other comment ____

Q24 Is there any particular element that you would like further information on regarding IBD ? (select as many as desired)

- Common symptoms of IBD
- Complications of IBD
- Causes of IBD
- Prognosis
- Risk of developing cancer
- How medications may affect me
- Nutrition and diet
- How to manage time away from work / school
- Sources of support for people with IBD
- How to connect with others with IBD
- Other comment ____-

Thank you for your time and participation in this survey.
